# SARS-CoV-2-specific antibody response characteristics in COVID-19 patients of different ages: SARS-CoV-2 antibody response in different aged patients

**DOI:** 10.3724/abbs.2022014

**Published:** 2022-02-17

**Authors:** Linfang Lu, Siqi Yu, Min Liu, Yang Li, Qing Lei, Mingxi Lin, Danyun Lai, Shujuan Guo, Hewei Jiang, Hongyan Hou, Yunxiao Zheng, Xuening Wang, Mingliang Ma, Bo Zhang, Hong Chen, Junbiao Xue, Hainan Zhang, Huan Qi, Ziyong Sun, Feng Wang, Xionglin Fan, Zhaowei Xu

**Affiliations:** 1 Key Laboratory of Gastrointestinal Cancer (Fujian Medical University) Ministry of Education Fuzhou 350122 China; 2 Laboratory of Scientific Research School of Basic Medical Sciences Fujian Medical University Fuzhou 350122 China; 3 Fujian Key Laboratory of Tumor Microbiology Department of Medical Microbiology Fujian Medical University Fuzhou 350122 China; 4 Shanghai Center for Systems Biomedicine Key Laboratory of Systems Biomedicine (Ministry of Education) Shanghai Jiao Tong University Shanghai 200240 China; 5 Department of Pathogen Biology School of Basic Medicine Tongji Medical College Huazhong University of Science and Technology Wuhan 430074 China; 6 Department of Clinical Laboratory Tongji Hospital Tongji Medical College Huazhong University of Science and Technology Wuhan 430074 China

**Keywords:** SARS-CoV-2, protein microarray, age, humoral immunity, IgG and IgM, non-structural/accessory protein

## Abstract

Age has been found to be one of the main risk factors for the severity and outcome of COVID-19. However, differences in SARS-CoV-2 specific antibody responses among COVID-19 patients of different age groups remain largely unknown. In this study, we analyzed the IgG/IgM responses to 21 SARS-CoV-2 proteins and 197 peptides that fully cover the spike protein against 731 sera collected from 731 COVID-19 patients aged from 1 to 92 years. We show that there is no overall difference in SARS-CoV-2 antibody responses in COVID-19 patients in the 4 age groups. By antibody response landscape maps, we find that the IgG response profiles of SARS-CoV-2 proteins are positively correlated with age. The S protein linear epitope map shows that the immunogenicity of the S-protein peptides is related to peptide sequence, disease severity and age of the COVID-19 patients. Furthermore, the enrichment analysis indicates that low S1 IgG responses are enriched in patients aged <50 and high S1 IgG responses are enriched in mild COVID-19 patients aged >60. In addition, high responses of non-structural/accessory proteins are enriched in severe COVID-19 patients aged >70. These results suggest the distinct immune response of IgG/IgM to each SARS-CoV-2 protein in patients of different age, which may facilitate a deeper understanding of the immune responses in COVID-19 patients.

## Introduction

COVID-19, which is caused by severe acute respiratory syndrome coronavirus 2 (SARS-CoV-2), has spread worldwide and evolved into a pandemic. SARS-CoV-2 has already caused 235,389,736 infections and claimed 4,808,886 lives as of October 5, 2021 ( https://coronavirus.jhu.edu/map.html) [1]. There is still no effective medicine-based treatment for COVID-19 [ 2, 3]. Humoral immune responses, especially SARS-CoV-2-specific antibody responses, play critical roles in disease progression, severity and final outcome. The understanding of SARS-CoV-2-specific antibody responses at the systems level is thus of great interest.

SARS-CoV-2 is a single-stranded RNA virus with an outer membrane envelope [4]. The genome of SARS-CoV-2 is ~29.9 kb and encodes 28 proteins, including 5 structural proteins, 15 non-structural proteins, and 8 accessory proteins [5]. The S protein consists of an N-terminal S1 fragment and a C-terminal S2 fragment [6], and it plays an essential role in viral attachment, fusion, and entry into the target cells that express the viral receptor, *i*. *e*., angiotensin-converting enzyme 2 [7]. Non-structural/accessory proteins of SARS-CoV-2 play an important role in virus replication and immune escape. For example, NSP1 inhibits host gene expression [8], NSP7, NSP8, and NSP12 form a complex and play an essential role in virus replication [9], and ORF9b inhibits type I IFN production [10]. Antibodies against the S protein and N protein are elicited in most patients, with higher titers in severe patients [11]. Additionally, 6 non-structural/accessory proteins, including NSP1, NSP7, NSP8, NSP12, ORF3b, and ORF9b, elicit strong antibody responses in COVID-19 patients [12]. Therefore, the clinical relevance of the antibody responses of SARS-CoV-2 proteins in COVID-19 patients has always been an important topic.

Age has been found to be the strongest risk factor for the severity and outcome of COVID-19 [ 13, 14]. The total antibody responses of SARS-CoV-2 are distinct in different age groups [15]. However, the features of SARS-CoV-2 specific immune responses against the S protein or non-structural/accessory proteins among COVID-19 patients of different ages remain largely unknown.

To explore the human antibody responses against SARS-CoV-2, we have constructed the SARS-CoV-2 proteome microarray containing 21 of 28 predicted proteins and 197 peptides of the spike protein [16]. In this study, by using this microarray, we screened 731 sera of COVID-19 patients, covering an age range of 1–92 years. Based on the data, we analyzed the features of SARS-CoV-2-specific antibody responses among COVID-19 patients of different age.

## Materials and Methods

### Patients and samples

In this study, 731 serum samples were collected from 731 COVID-19 patients admitted to Tongji Hospital of Wuhan from Jan 25, 2020 to Apr, 28, 2020. The COVID-19 patients were laboratory confirmed using the 7 ^th^ Diagnosis and Treatment Protocol for Novel Coronavirus Pneumonia. The severe cases met any one of the following criteria: (1) respiratory distress (≥30 breaths/min), (2) oxygen saturation ≤93% at rest, (3) arterial partial pressure of oxygen (PaO2)/fraction of inspired oxygen (FiO2) ≤300 mmHg (1 mmHg=0.133kPa), and (4) chest imaging that shows obvious lesion progression (>50%) within 24–48 h. All the serum samples were stored at –80°C until use. The study was approved by the Ethical Committee of Tongji Hospital, Huazhong University of Science and Technology (Wuhan, China) with No. ITJ-C20200128.

### Construction of SARS-CoV-2 proteome microarray

The protein microarray contained 21 of the 28 predicted SARS-CoV-2 proteins and 197 peptides derived from the spike protein. The spike protein was split to S1 (aa 1–685) and S2 (aa 686–1273) to increase protein expression. These 21 proteins were cloned into pGEX4-1 or pET32a vector and expressed in BL21 strain. The detailed protocol of protein purification was described by Jiang et al. [11] The detailed protocol of peptide preparation was described by Li *et al*. [ 17, 18]. Briefly, 197 peptides were derived from the spike protein (1273 aa) and produced by chemical synthesis, which are 13 aa in length with a cysteine at the N-terminus. The synthesized peptides were dissolved in PBS and conjugated to BSA protein to increase the stability. The 21 SARS-CoV-2 proteins and 197 spike protein peptides were printed into PATH® Protein Microarray Slides (GRACE, Oregon, USA) using a Super Marathon printer (Arrayjet, Edinburgh, UK).

### Serum analysis

The detailed protocol of microarray-based serum screening was described by Li *et al*. [ 12, 18]. Briefly, the protein microarray was blocked with 3% BSA buffer at 25°C for 3 h, and incubated with 200 μL diluted (1:200) serum at 25°C for 2 h. After 3 times wash with PBST, the microarray was incubated with anti-human IgG/IgM antibody at 25°C for 1 h with Cy3-conjugated and Alexa Fluor 647-conjugated, respectively (Jackson ImmunoResearch, Philadelphia, USA). After being washed with PBST for 3-times, the microarray was spin-dried at 25°C and scanned by LuxScan (CapitalBio, Beijing, China). The IgG and IgM signals were extracted with GenePix Pro 6.0 (Molecular Devices, San Jose, USA) and defined by foreground medians subtracted by background medians. The signal of a protein or peptide was averaged from triplicate spots.

### Quantification and statistical analysis

The data were analyzed using SPSS 24.0 and R i386 4.0.4 softwares. In antibody response intensity analysis, the IgG signals of 21 proteins were normalized by z-score in R to eliminate the signal difference of each protein. Uniform Manifold Approximation and Projection for Dimension Reduction assays were performed using the UMAP package in R with default parameters. The plot-blot was drawn using the ggplot2, reshape2, and RColorBrewer packages in R. The Pearson correlation coefficient was calculated using the dplyr, and tidyverse packages in R. The heatmap was constructed using the pheatmap, and tidyverse packages in R. The positive rate of antibody response for each protein was calculated in COVID-ONE database ( www.covid-one.cn) [19]. Comparisons between different groups were performed using chi-square tests for categorical variables and using two-sample t-tests for continuous variables in SPSS. Values of two-sided *P*<0.05 were considered to be statistically significant.

## Results

### SARS-CoV-2-specific antibody responses have no overall difference in patients of different ages

**Figure 1:**
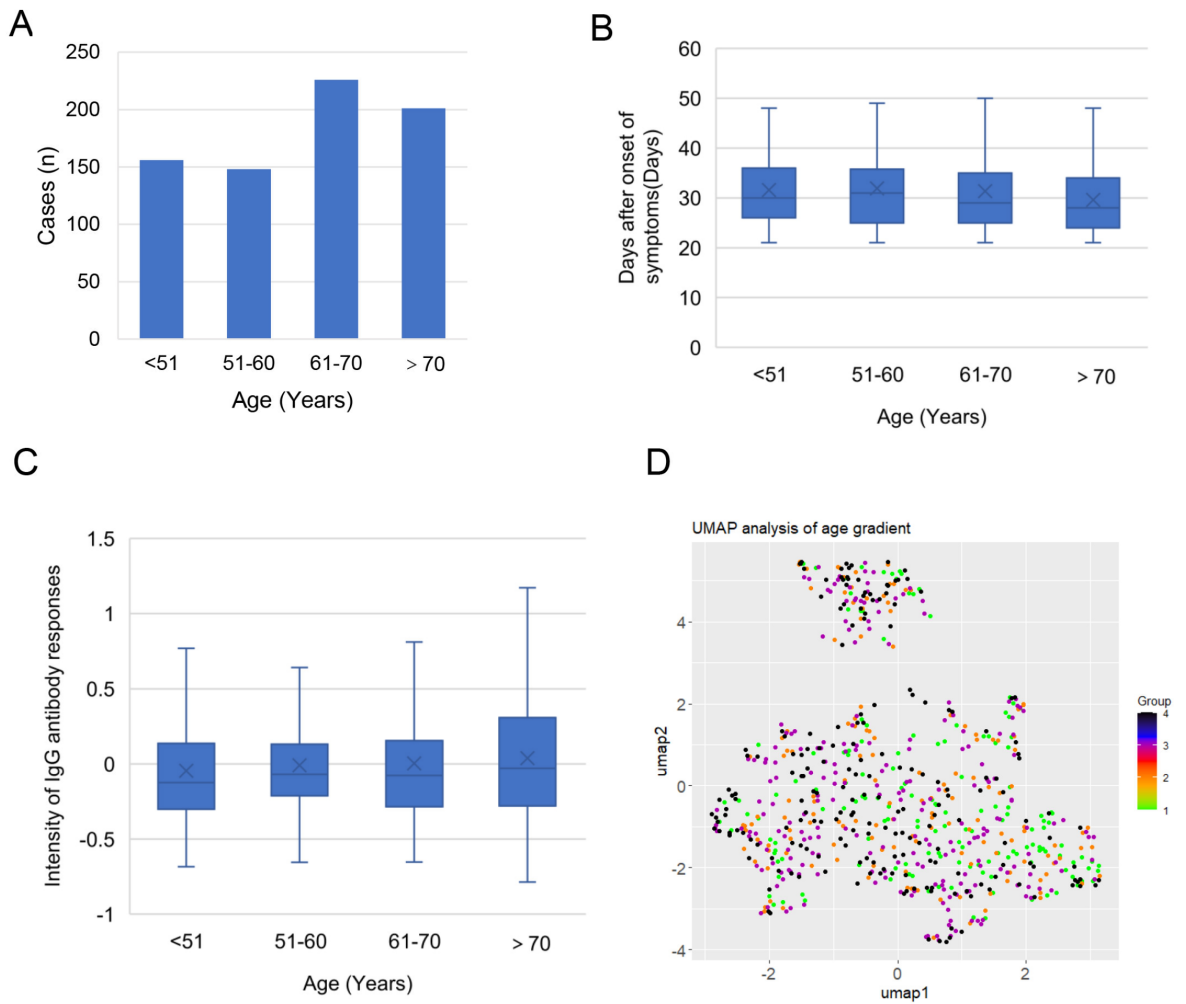
Figure1 The clinical information and overall SARS-CoV-2-specific antibody responses of COVID-19 patients (A) The age distribution of COVID patients in this study. (B) The time of onset to sampling for COVID patients of 4 age groups. (C) The average antibody intensity in the microarray for COVID patients of 4 age groups. (D) UMAP analysis for 731 COVID patients grouped by age.

A total of 731 serum samples from COVID-19 patients were collected in this study, which were at least 21 days after the onset of symptoms [20]. The patients included 362 males and 369 females, and the mean age was 63.8 years, with an age ranging from 1 to 92 years ( Table 1). We divided these patients into 4 groups by age, *i*. *e*., <51, 51–60, 61–70, and >70 ( Figure 1A), and the days after onset of symptoms of each group were not significantly different in these 4 age groups ( Figure 1B). The IgG responses of S1 and N peaked at 30 days after the onset of symptom and rapidly declined at 50 days after the onset of symptom ( Supplementary Figure S1A,B). The IgM responses of S1 and N peaked at 20 days after the onset of symptom and rapidly declined at 40 days after the onset of symptom ( Supplementary Figure S1C,D). The serum samples were collected at ~30 day after onset of symptoms ( Figure 1B), at which the IgM and IgG responses were relatively stable. To understand the risk of severe in COVID-19 patients of different ages, we performed the statistical comparison among the above groups. The results showed that Wald *χ* ^2^ of the binary logistic regression parameter for severity increased from 1 to 41.73 as the age increased, with the OR increased from 1 to 4.35 ( Table 2), indicating that age is a risk factor for the severity of COVID-19 patients, which is consistent with previous reports [13].

**Table:** **Table1** The clinical information of COVID-9 patients involved

Group		Case ( *n*)*
Gender	Male	362
Female	369
Severity	non-severe	336
Severe	395
Outcome	Survivor	679
Non-survivor	52

*The 731 patients were all from Tongji Hospital (Wuhan, China).

**Table:** **Table2** The clinical characteristics of diagnosed COVID-19 patients in different age groups

Age	Case ( *n*)	Mild ( *n*)	Severe ( *n*)	*β*	S.E.	Wald χ2	OR (95% CI)	*P*
<51	156	106	50				1	
51–60	148	68	80	0.89	0.24	14.21	2.44 (1.53, 3.89)	0.00
61–70	226	97	129	1.02	0.22	21.95	2.77 (1.81, 4.23)	0.00
>70	201	65	136	1.47	0.23	41.73	4.35 (2.78, 6.79)	0.00

To understand the response regularity of SARS-CoV-2-specific antibodies in COVID-19 patients, we screened 731 serum samples using a SARS-CoV-2 protein microarray for IgG and IgM responses. Thus, we obtained antibody response profiles against 21 SARS-CoV-2 proteins in 731 COVID-19 patients. To compare the overall SARS-CoV-2-specific antibody responses, we calculated the average of all antibody intensities in the microarray for each patient, and found that the antibody intensity was not significantly different among the 4 groups ( Figure 1C).

To further explore the antibody response in patients of different ages, we performed UMAP analysis, and the results showed that the 4 groups of patients were dispersedly distributed on the coordinate axis and had no significant clustering ( Figure 1D). These results suggest that there is no overall difference in SARS-CoV-2-specific antibody responses among patients of different ages.

### SARS-CoV-2-specific antibody response profile in COVID-19 patients of different ages

**Figure 2:**
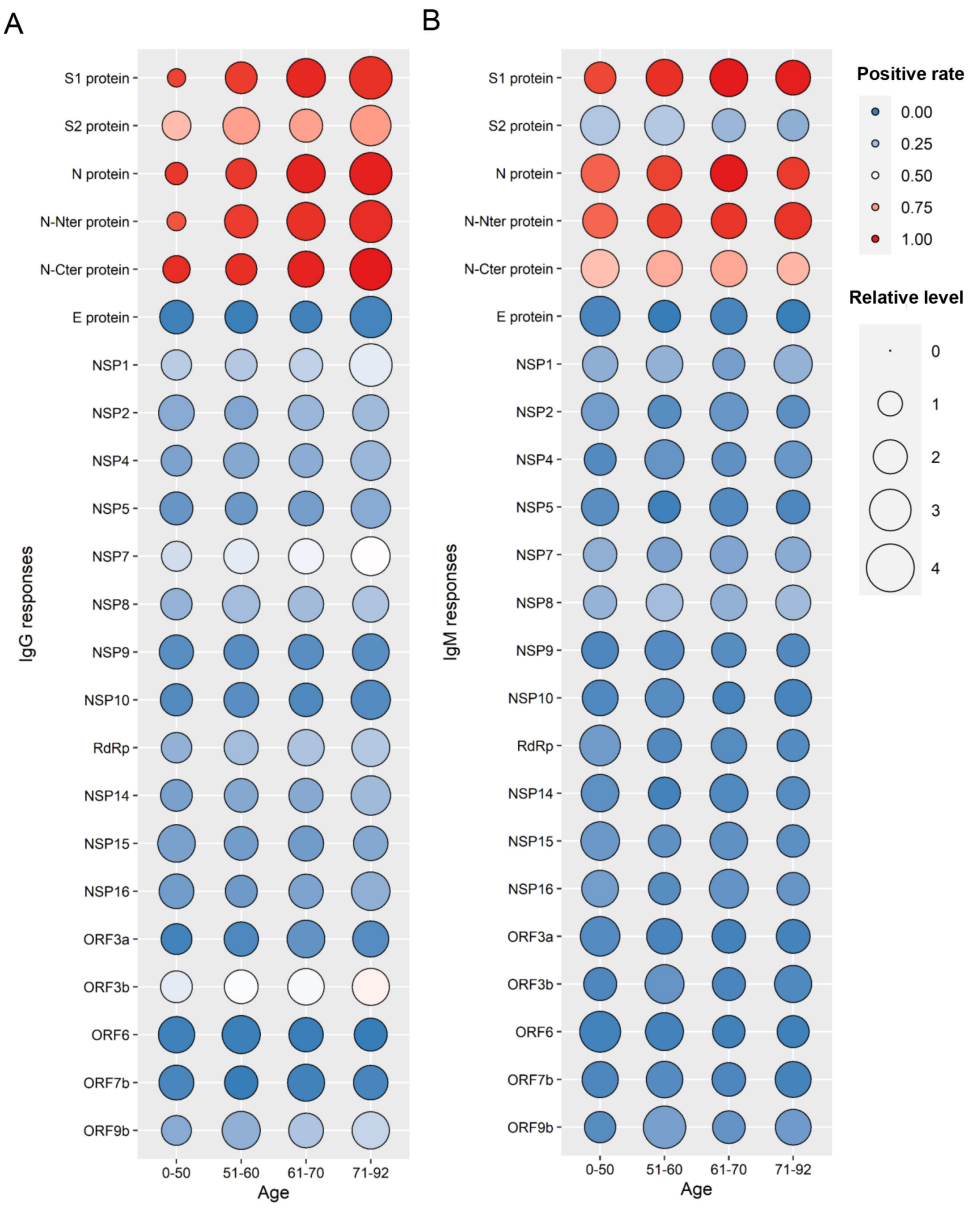
Figure2 The landscape of SARS-CoV-2 protein antibody responses in different age groups (A) IgG. (B) IgM.

To perform an in-depth analysis of the antibody responses of SARS-CoV-2 proteins, we constructed response landscape maps of IgG and IgM for 21 SARS-CoV-2 proteins ( Figure 2). Overall, with the exception of the NSP15 and ORF6 proteins, IgG responses were positively correlated with age and peaked at the age of 71–92. Conversely, IgM responses were not significantly different among the 4 age groups.

To understand the effect of disease severity on antibody response, we analyzed SARS-CoV-2 protein antibody responses for mild and severe COVID-19 patients respectively. The results showed that there were similar trends in antibody responses among different age groups, *i*. *e*., IgG responses were positively correlated with age ( Supplementary Figure S2) and IgM responses were not correlated with age ( Supplementary Figure S3). Hence, the IgG response profiles of SARS-CoV-2 proteins are positively correlated with ages.

### Antibody response profile of Spike-protein peptides in COVID-19 patients of different ages

**Figure 3:**
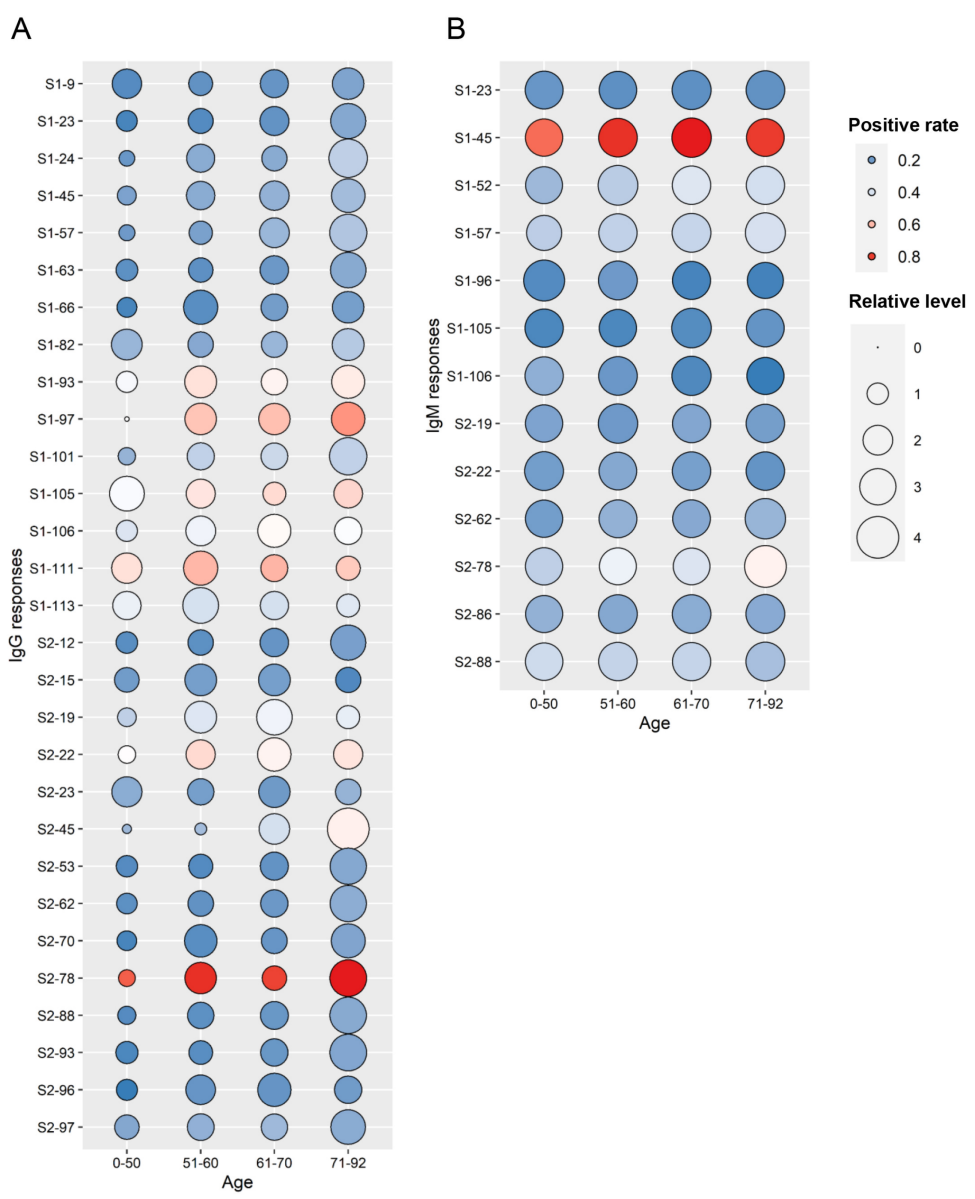
Figure3 The antibody response landscape of Spike-protein peptides in different age groups (A) IgG. (B) IgM.

The S protein of SARS-CoV-2 is known to play key role in the process of viral invasion to host cells. To understand the detailed immune characteristics of S protein, we constructed S protein linear epitope landscape for IgG and IgM responses in COVID-19 patients of different ages. The peptides with IgG or IgM positive rates over 10% were selected as the analysis object, so that 29 peptides for IgG and 13 peptides for IgM were included in the landscape map. The map showed that IgG responses of S protein peptides were positively correlated with age, except S1-105, S1-111, S1-113, S2-15, S2-19 and S2-22. But IgM responses were not significantly correlated with age ( Figure 3). The positive rates of S2-78 (aa 1148–1159) IgG response and S1-45 (aa 265–276) IgM response were close to 1 in patients aged 51–92, respectively ( Figure 3). These results suggest that S2-78 and S1-45 have higher immunogenicity and may play important roles in humoral immunity ( Figure 3A). Further, we analyzed the IgG/IgM antibody response landscape of Spike-protein peptides in mild and severe patients. The results showed that IgG responses were positively correlated with ages in severe patients but not in mild patients ( Supplementary Figure S4), and IgM responses were not correlated with disease severity ( Supplementary Figure S5). Hence, the immunogenicity of the S-protein peptides is related to peptide sequence, disease severity and age of the COVID-19 patients.

### Characterization of immune responses against S1 protein in COVID-19 patients of different ages

**Figure 4:**
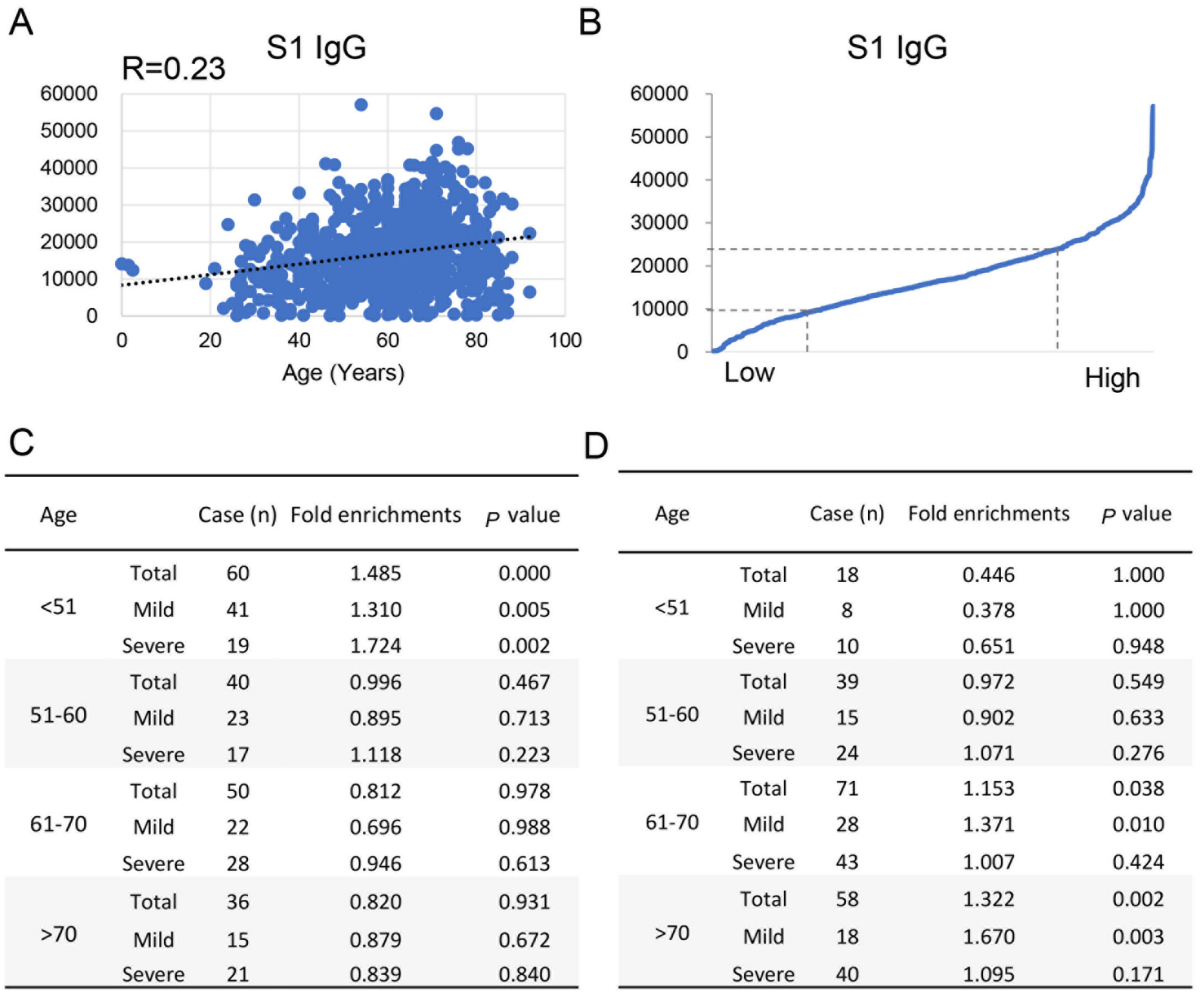
Figure4 Antibody responses of S1 protein in different age groups (A) Correlation analysis between S1 IgG response and age. (B) Signal distribution of S1 IgG response for 731 COVID-19 patients. (C) Enrichment statistical analysis for the patients with low S1 IgG responses. (D) Enrichment statistical analysis for the patients with high S1 IgG responses.

S1 IgG response is associated with SARS-CoV-2 neutralizing antibody titer [ 21, 22] and is an important index to evaluate the effectiveness of SARS-CoV-2 vaccine. Previous research has shown that S1 IgG response is positively correlated with age of the COVID-19 patients [23]. To further determine the characteristics of S1 protein IgG response in different age groups, we performed regression analysis of the S1 IgG response and age in COVID-19 patients, and found a positive correlation between S1 IgG response and age (R=0.23; Figure 4A). Then, according to the S1 protein IgG response signal, we divided the patients into three groups ( Figure 4B) with low, medium, high signals (25%, 50% and 25%, respectively), and calculated the enrichment of the three groups in different age. The results showed that low S1 IgG responses were enriched in the group of <50, but not correlated with disease severity ( Figure 4C), while high S1 IgG responses were enriched in mild COVID-19 patients in the group of >60 ( Figure 4D).

### Characterization of IgG responses against non-structural/accessory proteins in COVID-19 patients of different age

**Figure 5:**
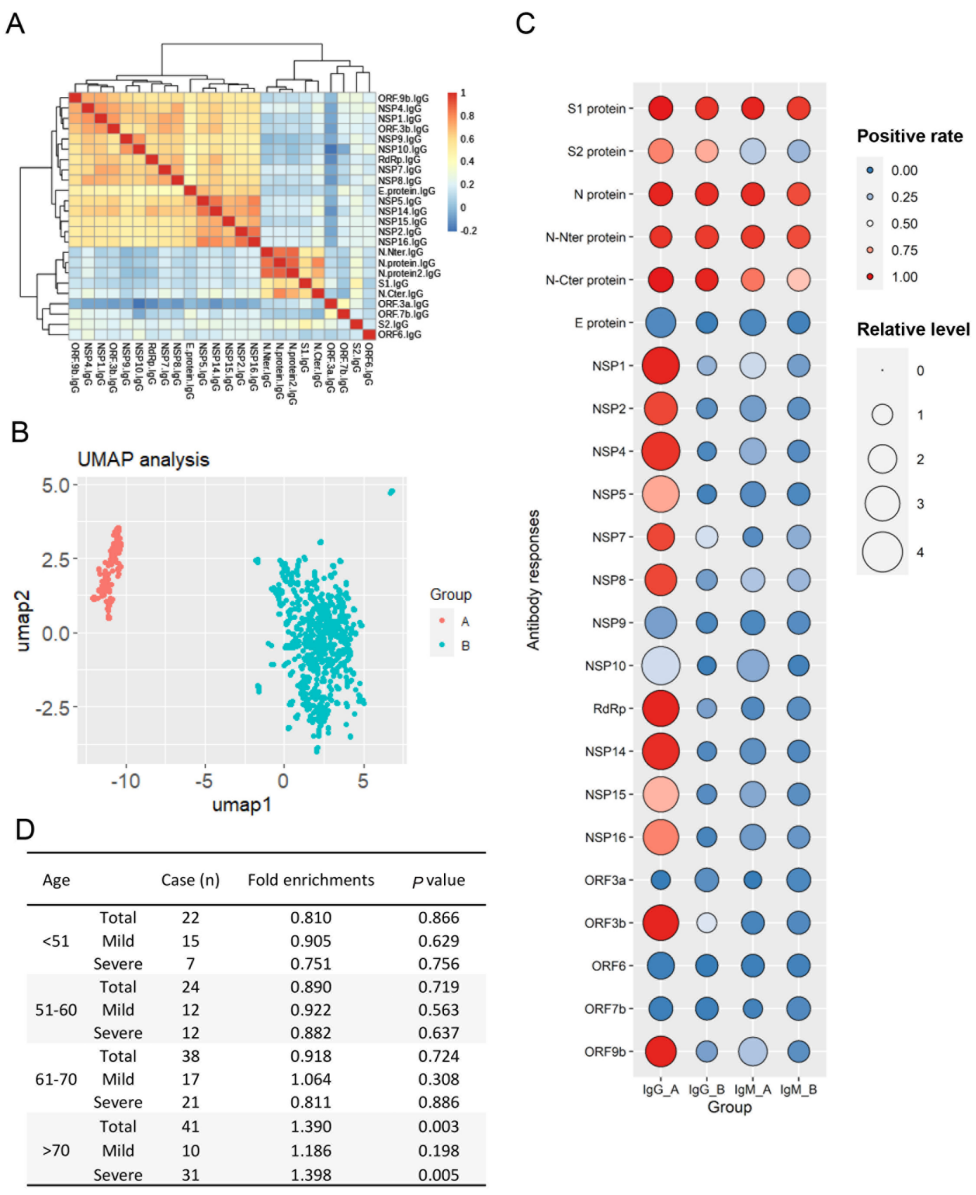
Figure5 Antibody responses of non-structural/accessory proteins in different age groups (A) Correlation analysis of IgG responses among 21 SARS-CoV-2 proteins. (B) UMAP analysis of 731 COVID-19 patients according to the IgG responses of non-structural/accessory proteins. (C) The plot shows the IgG/IgM responses of 21 SARS-CoV-2 proteins for Group A and Group B. (D) Enrichment statistical analysis for the patients with high IgG responses of non-structural/accessory proteins.

Non-structural/accessory proteins of SARS-CoV-2 play an important role in virus replication and immune escape [ 8– 10]. To explore the antibody response characteristics of SARS-CoV-2 non-structural/accessory proteins, we performed clustering analysis for IgG responses of SARS-CoV-2 proteins, and found that IgG responses of non-structural/accessory proteins are highly correlated, except ORF3a, ORF6 and ORF7b proteins ( Figure 5A). According the IgG responses of non-structural/accessory proteins, we performed UMAP assay for the 731 COVID-19 patients, and found that the patients were obviously clustered into two groups, with 125 patients in Group A and 606 patients in Group B ( Figure 5B). We displayed the antibody responses of SARS-CoV-2 proteins using plot diagram and found that the IgG/IgM responses of S1 and N proteins were not significantly different between Group A and Group B. Except ORF3a, ORF6 and ORF7b proteins, the IgG responses of non-structural/accessory proteins in Group A are higher than those in Group B ( Figure 5C). It is said that Group A patients had higher IgG responses of non-structural/accessory proteins than Group B patients. To explore the relevance between non-structural/accessory protein responses and age, we calculated the enrichment index of each age group in Group A, and the results showed that patients in the group of >70 with severe symptom were enriched in Group A ( Figure 5D). However, why high responses of non-structural/accessory proteins are associated with the severity of COVID-19 needs to be further explored.

## Discussion

By using a SARS-CoV-2 protein microarray, we generated SARS-CoV-2-specific global antibody response profiles and analyzed the SARS-CoV-2 antibody response characteristics in COVID-19 patients of different age. The results showed that there is no overall difference in SARS-CoV-2-specific antibody responses in patients of different age, but the IgG response profiles of SARS-CoV-2 proteins are distinct from IgM responses in COVID-19 patients of different ages. The S protein linear epitope map showed that the immunogenicity of the S-protein peptides is related to peptide sequence, disease severity and age of COVID-19 patients. Furthermore, the enrichment analysis indicated that low S1 IgG responses are enriched in COVID-19 patients aged <50 and high S1 IgG responses are enriched in mild COVID-19 patients aged >60. In addition, high responses of non-structural/accessory proteins are enriched in severe COVID-19 patients aged >70.

Previous studies have shown the association of age with SARS-CoV-2 antibody response by SARS-CoV-2 total antibody assay or RBD-targeted antibody assay [ 15, 24, 25]. By SARS-CoV-2 protein microarray, we constructed the antibody response landscape maps of 21 SARS-CoV-2 proteins, which showed the antibody response level of each protein in 4 age groups. We systematically compared the IgG and IgM responses of SARS-CoV-2 proteins and found that there are significant differences between IgG and IgM responses in COVID-19 patients of different age. Previous studies showed that IgG and IgM responses peaked at 6 weeks and 4 weeks respectively after the onset of symptoms [ 26, 27], proving that IgG and IgM have different responses in different onset time. In this study, we for the first time showed the association of age with IgG/IgM responses of SARS-CoV-2 proteins.

Non-structural/accessory proteins play the important roles in virus replication and immune escape [ 8– 10] and the IgG responses of non-structural/accessory proteins are distinct from that of S1 and N proteins [12]. The correlation between antibody responses of non-structural/accessory proteins and age is an interesting topic. By correlation and UMAP analysis, we found that the population with high responses of non-structural/accessory proteins were enriched in severe COVID-19 patients aged >70. These results indicate that high responses of non-structural/accessory proteins are enriched in severe COVID-19 patients aged >70. Interestingly, high IgG responses of S1 protein are enriched in mild COVID-19 patients aged >70. It is said that the non-structural/accessory proteins and spike protein may have different functions in the process of virus pathogenicity [ 28, 29].

The specific immunities of SARS-CoV-2 proteins have partial similarity in COVID-19 patients and the people injected with inactivated or attenuated SARS-CoV-2 vaccine [22]. In this study, we provide a comprehensive antibody profile for different age, which may facilitate an in-depth understanding of the humoral immunity of COVID-19 patients as well as the vaccinated volunteers at the systemic level.

## Supplementary Data

Supplementary data is available at *Acta Biochimica et Biophysica Sinica* online.

## Supporting information

21568Supplementary_Figures

## References

[1] Dong E, Du H, Gardner L (2020). An interactive web-based dashboard to track COVID-19 in real time. Lancet Infect Dis.

[2] Knipe DM, Levy O, Fitzgerald KA, Mühlberger E (2020). Ensuring vaccine safety. Science.

[3] Lipsitch M, Dean NE (2020). Understanding COVID-19 vaccine efficacy. Science.

[4] Finkel Y, Mizrahi O, Nachshon A, Weingarten-Gabbay S, Morgenstern D, Yahalom-Ronen Y, Tamir H (2021). The coding capacity of SARS-CoV-2. Nature.

[5] Wu A, Peng Y, Huang B, Ding X, Wang X, Niu P, Meng J (2020). Genome composition and divergence of the novel coronavirus (2019-nCoV) originating in China. Cell Host Microbe.

[6] Wrapp D, Wang N, Corbett KS, Goldsmith JA, Hsieh CL, Abiona O, Graham BS (2020). Cryo-EM structure of the 2019-nCoV spike in the prefusion conformation. Science.

[7] Lan J, Ge J, Yu J, Shan S, Zhou H, Fan S, Zhang Q (2020). Structure of the SARS-CoV-2 spike receptor-binding domain bound to the ACE2 receptor. Nature.

[8] Thoms M, Buschauer R, Ameismeier M, Koepke L, Denk T, Hirschenberger M, Kratzat H (2020). Structural basis for translational shutdown and immune evasion by the Nsp1 protein of SARS-CoV-2. Science.

[9] Yin W, Mao C, Luan X, Shen DD, Shen Q, Su H, Wang X (2020). Structural basis for inhibition of the RNA-dependent RNA polymerase from SARS-CoV-2 by remdesivir. Science.

[10] Jiang HW, Zhang HN, Meng QF, Xie J, Li Y, Chen H, Zheng YX (2020). SARS-CoV-2 Orf9b suppresses type I interferon responses by targeting TOM70. Cell Mol Immunol.

[11] Jiang HW, Li Y, Zhang HN, Wang W, Yang X, Qi H, Li H (2020). SARS-CoV-2 proteome microarray for global profiling of COVID-19 specific IgG and IgM responses. Nat Commun.

[12] Li Y, Xu Z, Lei Q, Lai DY, Hou H, Jiang HW, Zheng YX (2021). Antibody landscape against SARS-CoV-2 reveals significant differences between non-structural/accessory and structural proteins. Cell Rep.

[13] Zhang X, Tan Y, Ling Y, Lu G, Liu F, Yi Z, Jia X (2020). Viral and host factors related to the clinical outcome of COVID-19. Nature.

[14] Liu Y, Mao B, Liang S, Yang JW, Lu HW, Chai YH, Wang L (2020). Association between ages and clinical characteristics and outcomes of coronavirus disease 2019. Eur Respir J.

[15] Yang HS, Costa V, Racine-Brzostek SE, Acker KP, Yee J, Chen Z, Karbaschi M (2021). Association of age with SARS-CoV-2 antibody response. JAMA Netw Open.

[16] Li Y, Lai DY, Zhang HN, Jiang HW, Tian X, Ma ML, Qi H (2020). Linear epitopes of SARS-CoV-2 spike protein elicit neutralizing antibodies in COVID-19 patients. Cell Mol Immunol.

[17] Li Y, Ma ML, Lei Q, Wang F, Hong W, Lai DY, Hou H (2021). Linear epitope landscape of the SARS-CoV-2 Spike protein constructed from 1,051 COVID-19 patients. Cell Rep.

[18] Li Y, Lai DY, Tao SC (2021). SARS-CoV-2 Spike linear epitope scanning via a peptide microarray through sera profiling. STAR Protocols.

[19] Xu Z, Li Y, Lei Q, Huang L, Lai DY, Guo SJ, Jiang HW, et al. COVID-ONE-humoral immune: the one-stop database for COVID-19-specific antibody responses and clinical parameters. bioRxiv 2021. Doi: 10.1101/2021.07.29.454261.

[20] WHO. WHO Laboratory testing for 2019 novel coronavirus (2019-nCoV) in suspected human cases. 2020.

[21] Schlickeiser S, Schwarz T, Steiner S, Wittke K, Al Besher N, Meyer O, Kalus U (2020). Disease severity, fever, age, and sex correlate with SARS-CoV-2 neutralizing antibody responses. Front Immunol.

[22] Ma ML, Shi DW, Li Y, Hong W, Lai DY, Xue JB, Jiang HW (2021). Systematic profiling of SARS-CoV-2-specific IgG responses elicited by an inactivated virus vaccine identifies peptides and proteins for predicting vaccination efficacy. Cell Discov.

[23] Glück V, Grobecker S, Tydykov L, Salzberger B, Glück T, Weidlich T, Bertok M (2021). SARS-CoV-2-directed antibodies persist for more than six months in a cohort with mild to moderate COVID-19. Infection.

[24] Klein SL, Pekosz A, Park HS, Ursin RL, Shapiro JR, Benner SE, Littlefield K (2020). Sex, age, and hospitalization drive antibody responses in a COVID-19 convalescent plasma donor population. J Clin Investigation.

[25] Richards NE, Keshavarz B, Workman LJ, Nelson MR, Platts-Mills TAE, Wilson JM (2021). Comparison of SARS-CoV-2 antibody response by age among recipients of the BNT162b2 vs the mRNA-1273 vaccine. JAMA Netw Open.

[26] Wu J, Liang B, Chen C, Wang H, Fang Y, Shen S, Yang X (2021). SARS-CoV-2 infection induces sustained humoral immune responses in convalescent patients following symptomatic COVID-19. Nat Commun.

[27] Long QX, Liu BZ, Deng HJ, Wu GC, Deng K, Chen YK, Liao P (2020). Antibody responses to SARS-CoV-2 in patients with COVID-19. Nat Med.

[28] Redondo N, Zaldívar-López S, Garrido JJ, Montoya M (2021). SARS-CoV-2 accessory proteins in viral pathogenesis: knowns and unknowns. Front Immunol.

[29] Yadav R, Chaudhary JK, Jain N, Chaudhary PK, Khanra S, Dhamija P, Sharma A (2021). Role of structural and non-structural proteins and therapeutic targets of SARS-CoV-2 for COVID-19. Cells.

