# Supplementary material for: SARS-CoV-2-specific antibody response characteristics in COVID-19 patients of different ages: SARS-CoV-2 antibody response in different aged patients
Source: Acta Biochim Biophys Sin (Shanghai). 2022 Feb 17;54(4):556–64. doi: 10.3724/abbs.2022014 (PMC9828714; doi:10.3724/abbs.2022014)
Supplement: 21568Supplementary_Figures [file 21568Supplementary_Figures.docx]

**
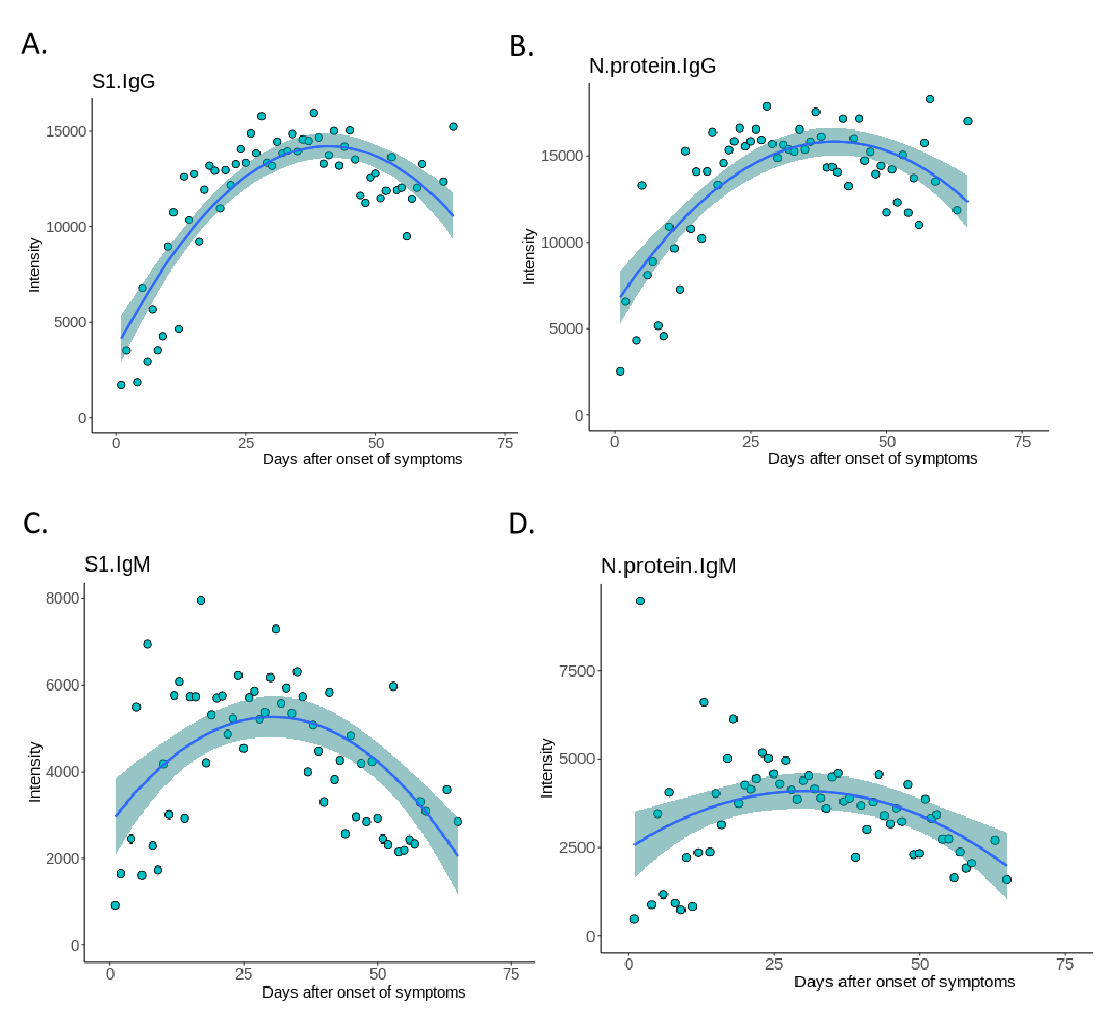
**

**Supplementary Figure S1. Dynamic antibody responses to S1 and N proteins** Scatter plot showing dynamic antibody responses to S1 IgG (A), N protein IgG (B), S1 IgM (C), and N protein IgM (D).

**
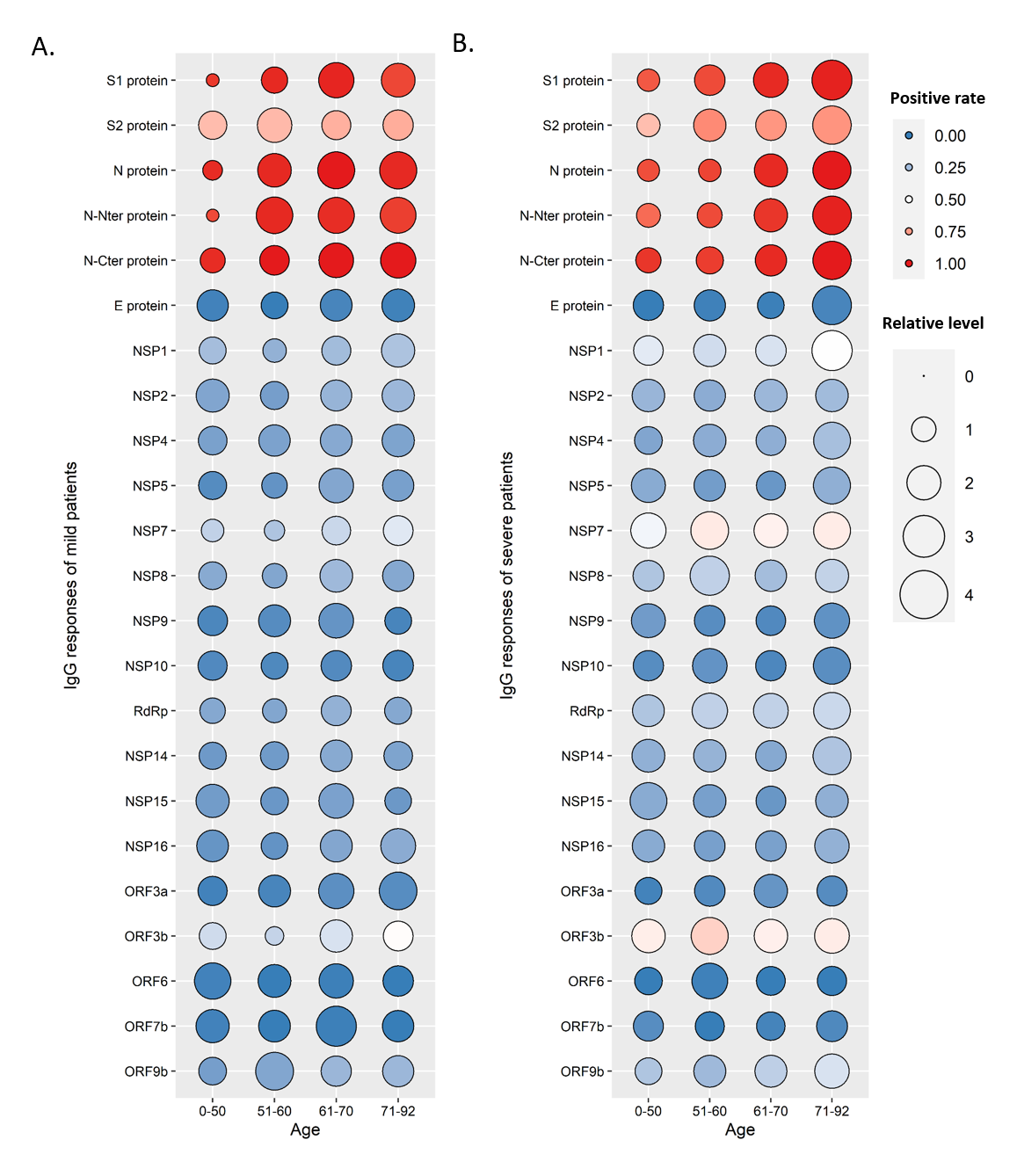
**

**Supplementary Figure S2. The landscape of SARS-CoV-2 protein IgG antibody responses in mild (A) and severe (B) patients**

**
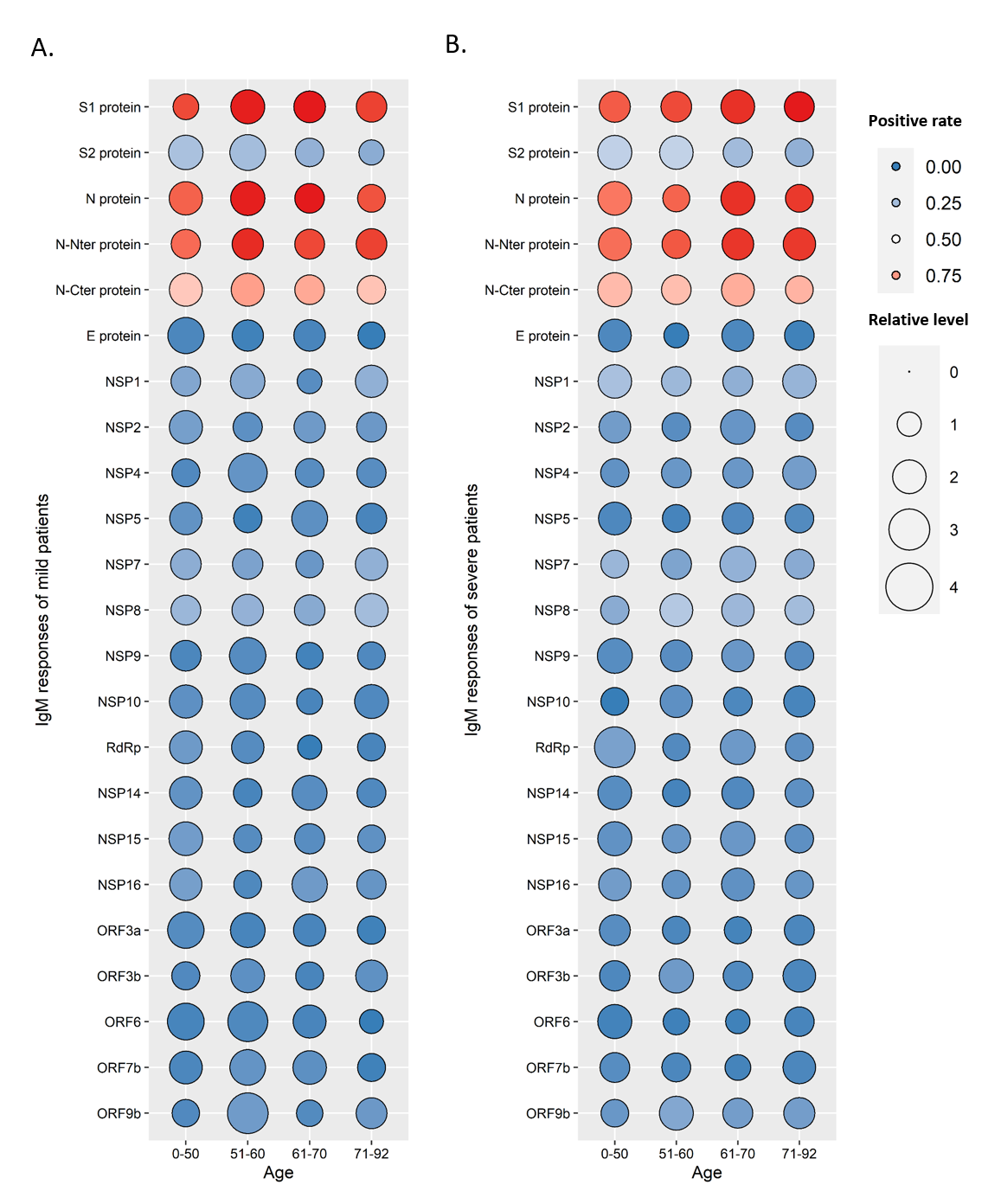
**

**Supplementary Figure S3. The landscape of SARS-CoV-2 protein IgM antibody responses in mild (A) and severe (B) patients**

**
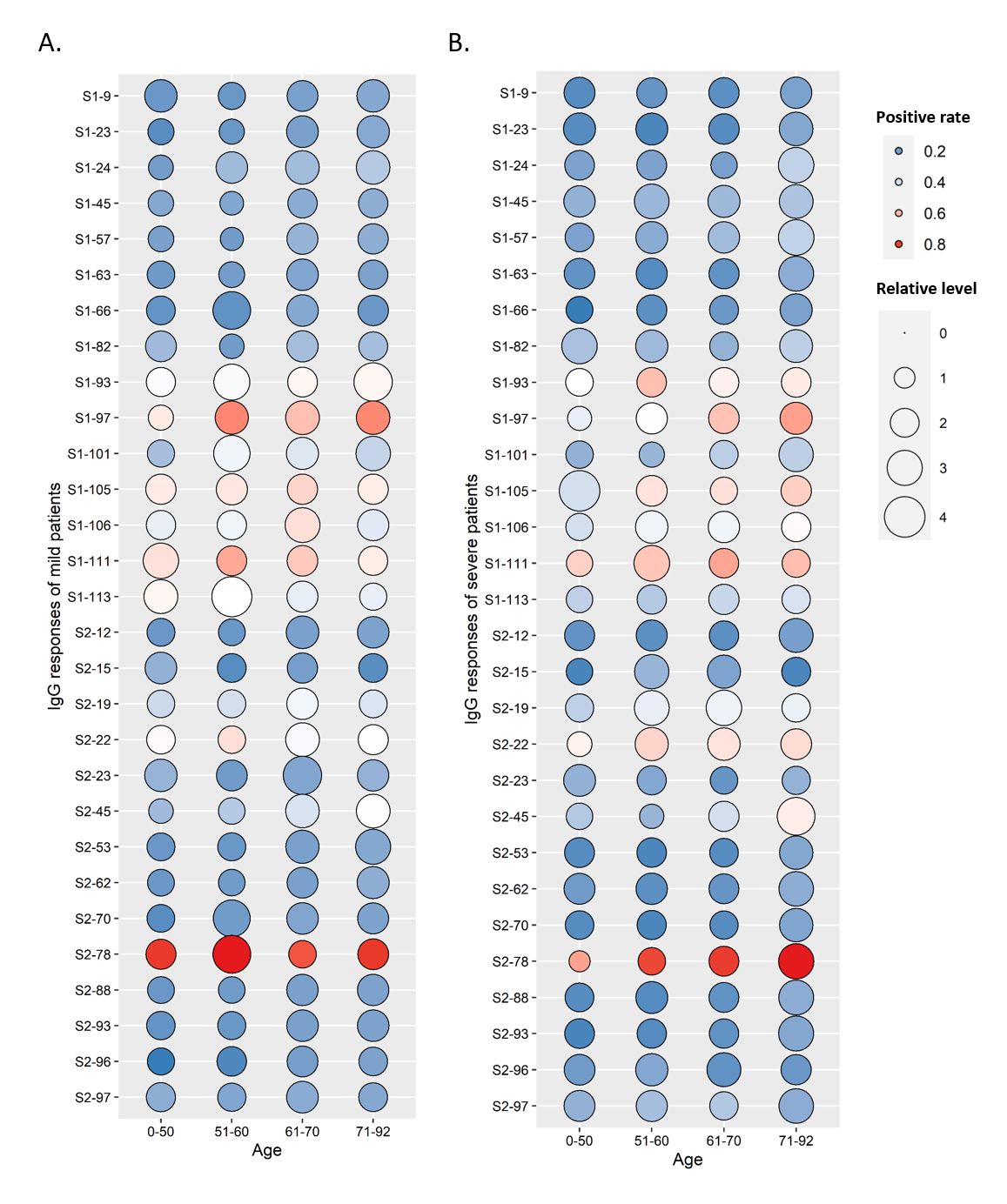
**

**Supplementary Figure S4. The IgG antibody response landscape of Spike-protein peptides in mild (A) and severe (B) patients**

**
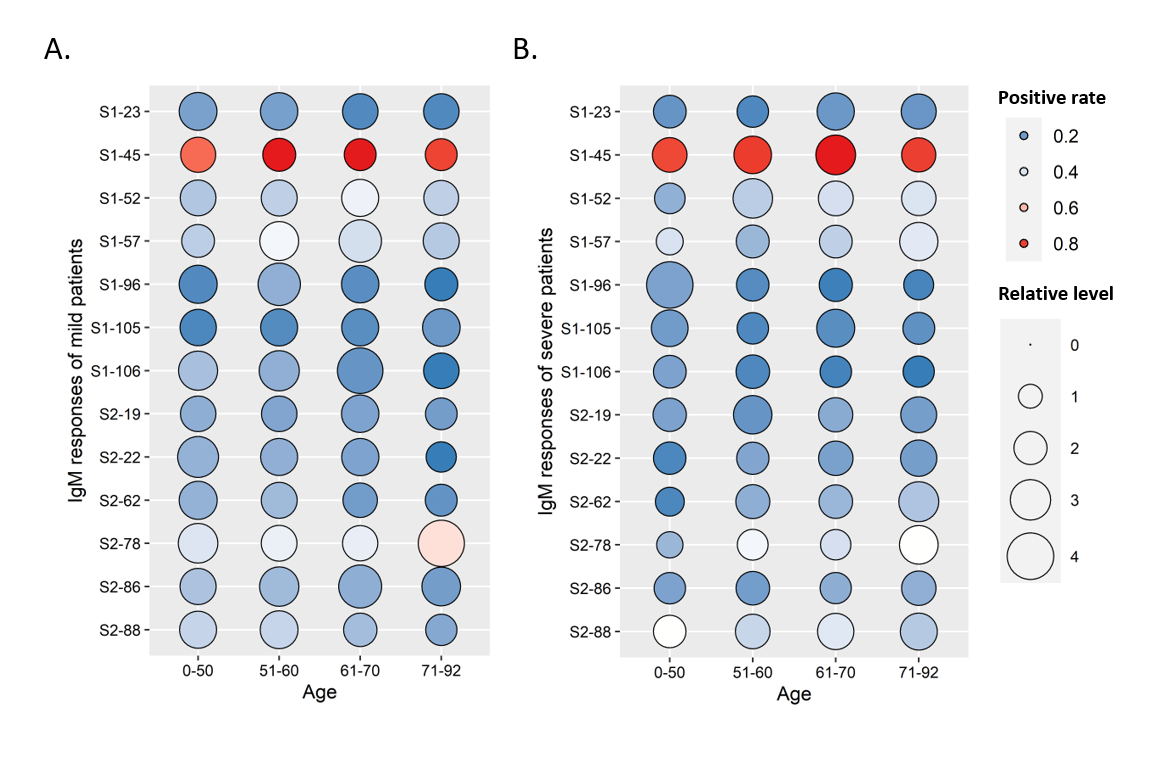
**

**Supplementary Figure S5. The IgM antibody response landscape of Spike-protein peptides in mild (A) and severe (B) patients**
